# Atrial Myxoma Presenting as Positional Vertigo: A Case Report

**DOI:** 10.7759/cureus.4884

**Published:** 2019-06-11

**Authors:** Aamani Jupalli, Ateeq Mubarik, Arshad Muhammad Iqbal, Mowaffak Atfeh, Salman Muddassir

**Affiliations:** 1 Internal Medicine, Oak Hill Hospital, Brooksville, USA; 2 Internal Medicine, Ascension St. Michael's Hospital, Stevens Point, USA; 3 Cardiology, Oak Hill Hospital, Brooksville, USA

**Keywords:** myxoma, vertigo, atrial myxoma, syncope

## Abstract

Cardiac myxoma is an uncommon diagnosis but presents with common manifestations. There is a wide range of symptomatology from non-specific fever and weight loss to stroke-like symptoms. It is also one of the rare causes of cardiac syncope and thromboembolic events. We present a case of a 67-year-old female who presented with seemingly benign vertigo symptoms which she ignored for years before seeking medical attention. An echocardiogram revealed a 3.5 x 3.0 x 1.0 cm atrial myxoma which was surgically excised. Her symptoms have resolved entirely with no recurrence since surgery.

## Introduction

Cardiac myxoma is a benign cardiac tumor diagnosed in adults and accounts for over 50% of all cardiac tumors [1]. The tumor originates from subendothelial multipotent mesenchymal heart cells [2]. In 60%-80% of cases, myxomas are found in the left atrium (LA) and are typically inserted in the interatrial septum close to fossa ovalis. However, they are also found in other locations of the left atrium, right atrium, ventricles, and valves [3]. Depending on their location, myxomas can mimic different clinical manifestations like mitral stenosis, subacute infective endocarditis or vasculitis. Fever is the most common manifestation in young patients. Other more common presentations include three kinds of symptoms: (i) obstructive, relating to obstruction of the mitral valve, (ii) embolic, most frequently cerebral; and (iii) systemic, involving non-specific features such as fever or tiredness [4]. Our case describes a patient who presents with persistent dizziness that leads to the discovery of a cardiac tumor.

## Case presentation

**Figure 1:**
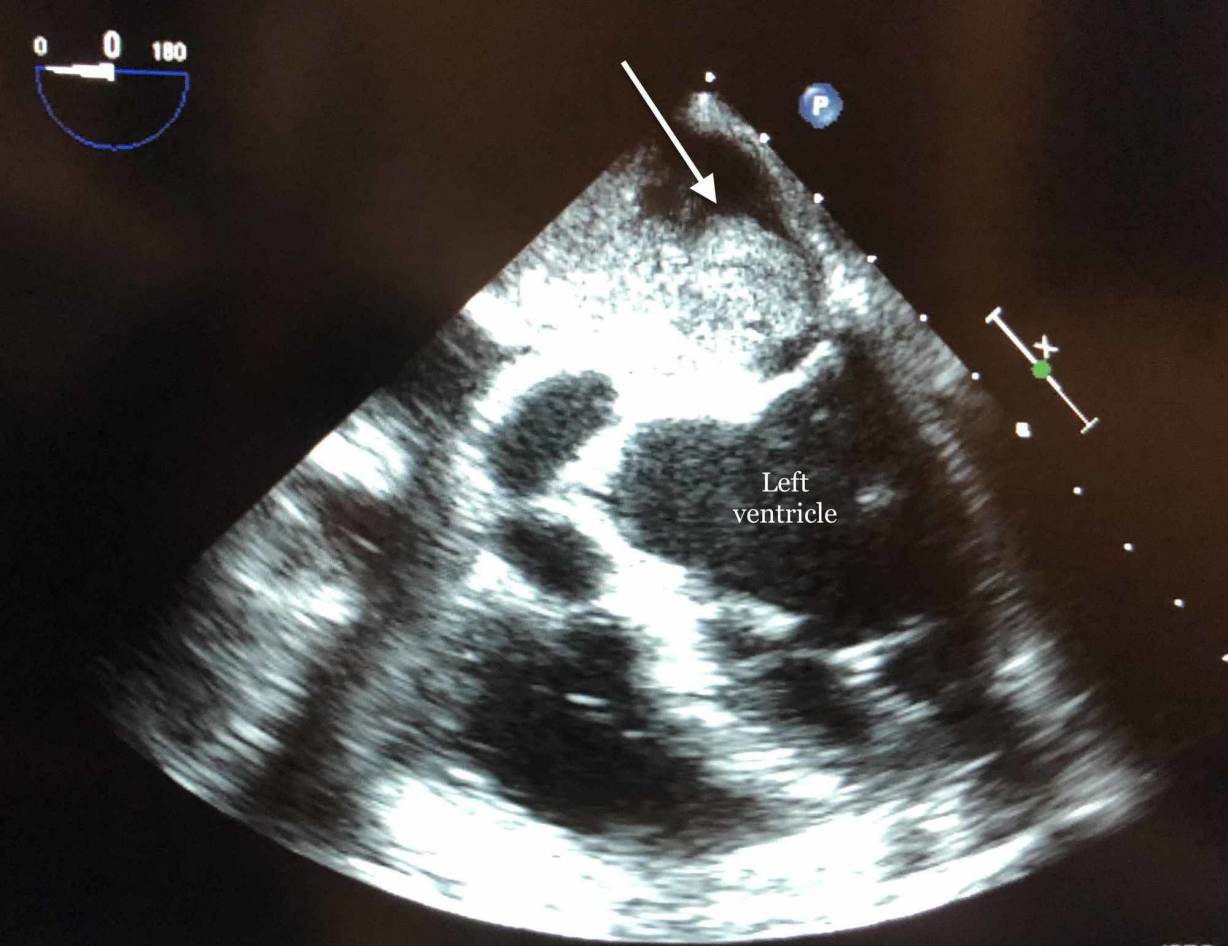
Transesophageal echocardiogram depicting left atrial myxoma (white arrow).

**Figure 2:**
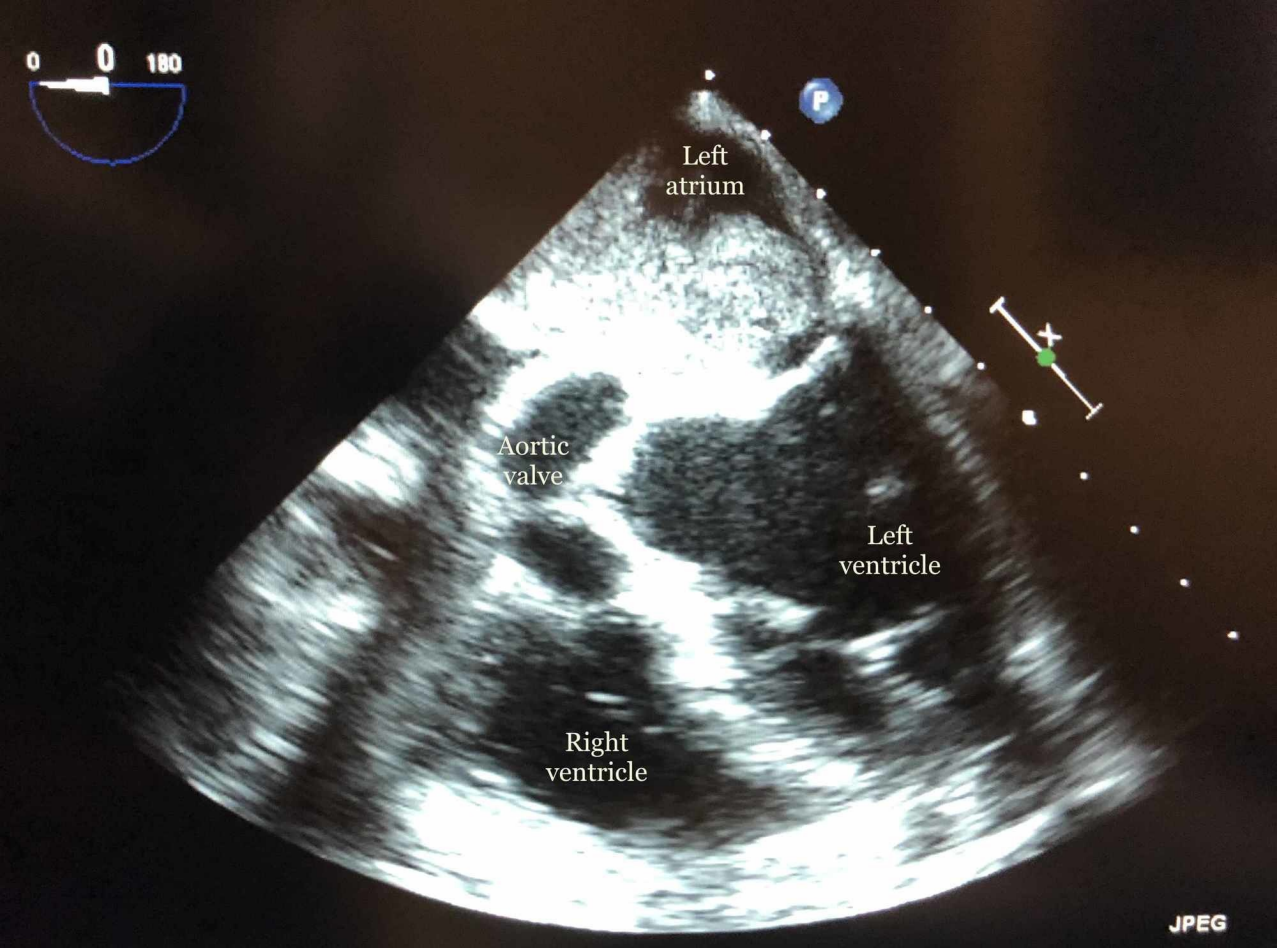
Areas of the heart visible from a transesophageal echocardiogram with myxoma in the left atrium.

A 67-year-old female with a significant past medical history of generalized anxiety disorder and supraventricular tachycardia, which was controlled on a beta-blocker, initially presented in an outpatient setting with complaints of dizziness. The symptoms initially started two years ago but she did not seek medical attention as they were not interfering with her day to day activities. She used to follow up with her primary care physician and was initially prescribed meclizine. Two weeks before hospital admission, her symptoms became progressively worse and persistent. An MRI was ordered by her primary care physician which revealed an acute right parietal and subacute left parietal cerebrovascular accident (CVA). She was referred to the hospital to evaluate the etiology of her stroke. On admission, she described the dizziness as triggered and continuous with increasing symptoms upon bending forward. She denied nausea, vomiting, tinnitus, sweating, fever or a history of recent infections. She also has a history of shortness of breath with moderate to heavy activities, however, denied active chest pain, palpitations or history of atrial fibrillation. Records from her primary care physician revealed a carotid ultrasound which was unremarkable for significant bilateral carotid artery stenosis. Her vital signs were stable on admission. A cardiovascular examination revealed soft S1 and S2 with diastolic murmur over the apex. The rest of the physical examination including orthostatic vitals were negative. Initial laboratory workups including complete blood count (CBC), complete metabolic panel (CMP) and chest X-ray (CXR) were within normal limits. Electrocardiogram revealed sinus rhythm with left axis deviation and poor R-wave progression from V1 to V3. Cardiology was consulted for the evaluation of her shortness of breath and dizziness. An echocardiogram was performed which revealed a large, lobulated mass most consistent with atrial myxoma with normal left ventricular systolic and diastolic function. The mass was occupying about half of the left atrium prolapsing through the mitral valve partially into the left ventricle as depicted in Figures 1-2.

**Figure 3:**
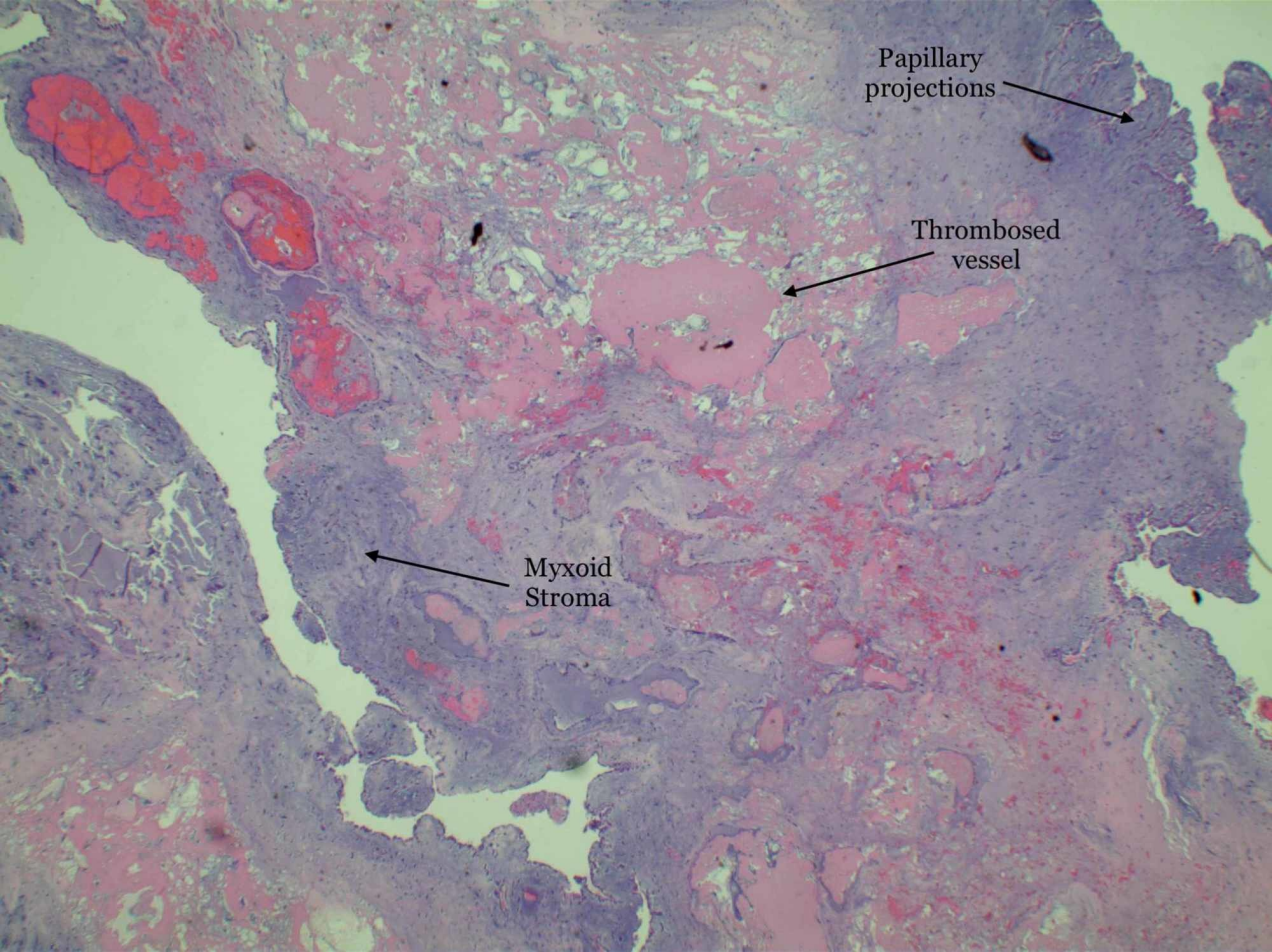
Low power magnification showing an abundance of mucopolysaccharide matrix of myxoid stroma mixed with papillary projections and stellate cells mixed with blood vessels.

**Figure 4:**
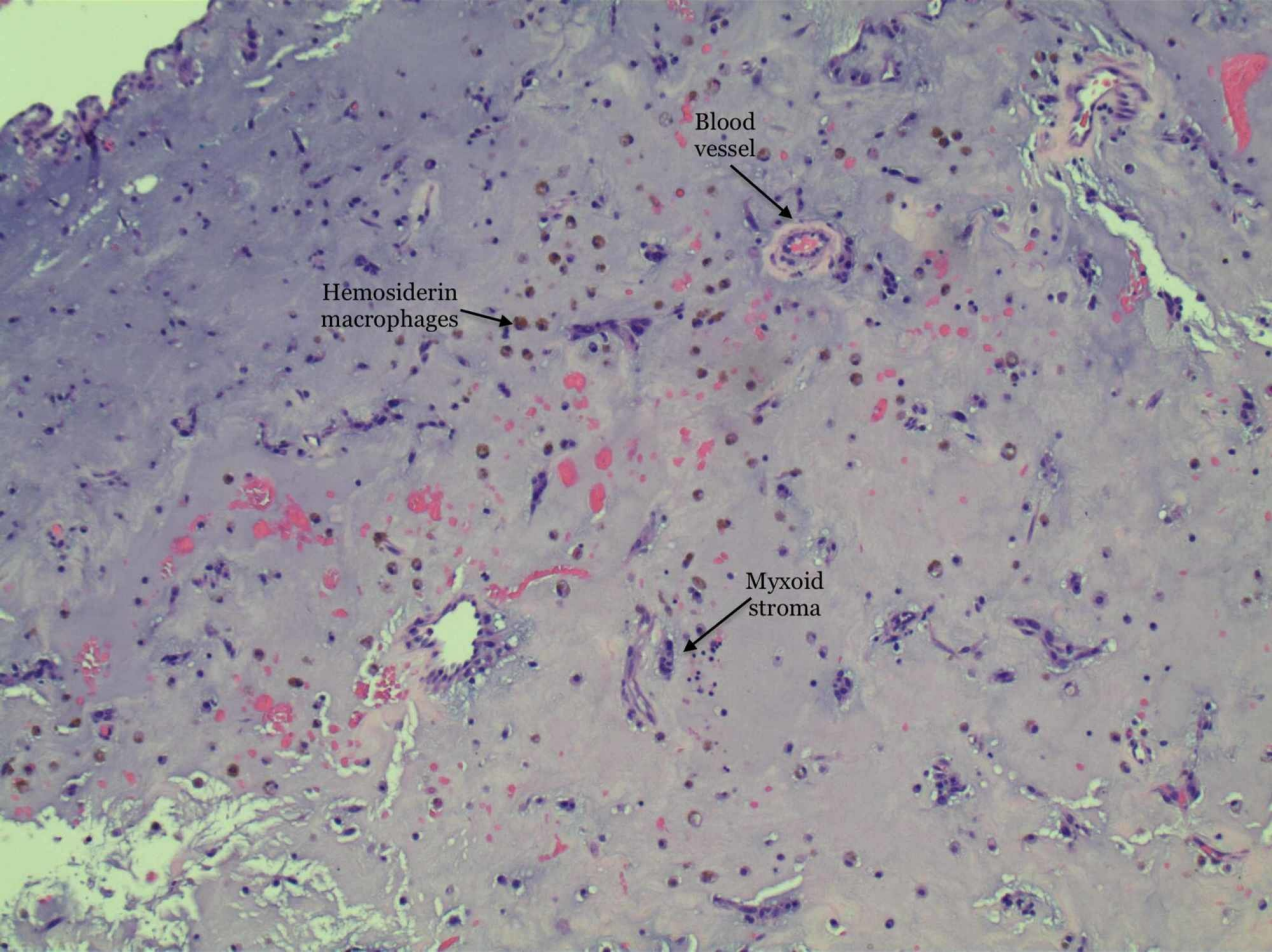
High power magnification showing hemosiderin macrophages with islands of stellate cells.

**Figure 5:**
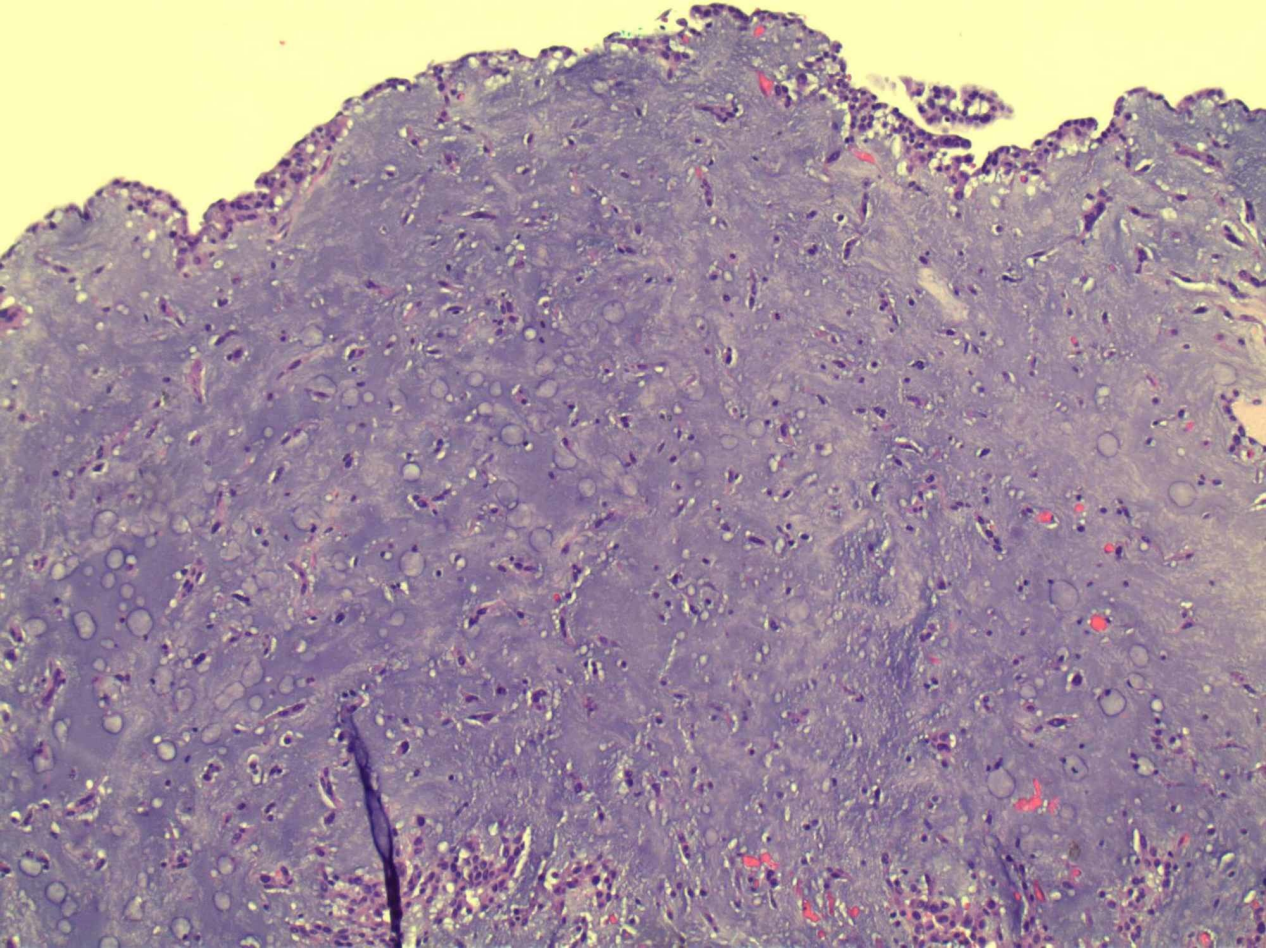
Diffuse spindle-shaped stroma with mixed myxoid cells.

Patent foramen ovale was ruled out with the help of a transesophageal echocardiogram. The plan was to remove the myxoma suspecting stroke as the likely etiology of her symptoms. She underwent cardiac catheterization prior to surgery to rule out concomitant coronary artery disease which showed no evidence of significant stenosis. The patient subsequently underwent removal of atrial myxoma as well as left atrial appendage ligation. Histopathological analysis of the specimen confirmed the diagnosis of benign myxoma measuring 3.5 x 3.0 x 1.0 cm. Biopsy of left atrial appendage muscle showed benign cardiac muscle with no necrosis or thrombi. Pathology slides are illustrated in Figures 3-5.

Her symptoms completely resolved after the surgery and she was discharged with a scheduled follow-up with her primary care physician. The patient was then followed up for six months and did not report the recurrence of symptoms since the time of surgery.

## Discussion

Atrial myxoma is the most common benign cardiac tumor [5] representing 50-75% of all cardiac tumors [6]. The mass can be mild or metastatic. Symptoms vary from nonspecific to a wide range of systemic manifestations including but not limited to fever, weight loss, myalgias, and arthralgias [7]. If clinical suspicion for subacute infective endocarditis is low, atrial myxoma should be considered as a differential diagnosis [8]. Cardiac symptoms include exertional shortness of breath, palpitations or syncope [7].

In some cases, it can present with stroke-like symptoms as its initial manifestation [9]. Although the most common site of atrial myxoma is the left atrium, it can arise from the right atrium [10]. Pathogenesis includes inflammation as the primary mechanism where lepidic cells in the atrial myxoma release cytokines such as interleukin (IL)-6, which attract inflammatory cells and trigger a systemic inflammatory response to stimulate angiogenesis and tumor growth [9]. What is heard on cardiovascular examination depends on the site of myxoma. Usually, in LA myxoma, the first heart sound (S1) becomes more intense because of functional mitral stenosis due to the movement of the tumor across the valve [11]. This also gives tumor plop and diastolic rumble murmur such as in our patient. Our patient presented with persistent, non-specific vertigo symptoms which improved with removal of the myxoma. Although the clinical picture might suggest a benign etiology, the clinician should consider a broad range of differential diagnoses including atrial myxoma like in the case described above.

Laboratory analysis can reveal elevated inflammation markers, leukocytosis, thrombocytosis or thrombocytopenia and normocytic anemia which is why it is essential to differentiate myxoma from other chronic inflammatory conditions such as subacute infective endocarditis [12]. Echocardiography is the most common imaging study to diagnose a cardiac myxoma and to differentiate from other causes. Vegetation can easily be seen on an echocardiogram if it is significant and on the right side of the heart. Echocardiography will locate the myxoma but determining whether it is benign or malignant will require further testing [13]. A CT or MRI can be used to demarcate the mass further [14]. Cardiac biopsy is not usually needed, but if malignancy is suspected, then the biopsy should be done after surgical excision [12].

The gold standard treatment is surgical excision if symptomatic, however benign symptoms such as the ones present in our patient do not usually require surgical removal [15]. In our case, the MRI brain showed multiple parietal ischemic infarcts, so surgery was done to prevent further strokes. Surprisingly, the symptoms of vertigo resolved after surgery indicating that the myxoma was the cause of persistent symptoms. The patient was being monitored in an outpatient setting for the recurrence of symptoms, and after six months of follow-up, no recurrence of symptoms was reported by the patient.

## Conclusions

To conclude, cardiac myxoma is a benign diagnosis and is an easily treatable disease. There should be a high degree of suspicion if the patient presents with stroke-like symptoms. However, in a few cases such as the one presented above, the seemingly benign symptoms of persistent dizziness can be a manifestation of an underlying myxoma which should not be ignored.
